# Experimental demonstration of a parasite-induced immune response in wild birds: Darwin's finches and introduced nest flies

**DOI:** 10.1002/ece3.651

**Published:** 2013-06-26

**Authors:** Jennifer A H Koop, Jeb P Owen, Sarah A Knutie, Maria A Aguilar, Dale H Clayton

**Affiliations:** 1 Department of Biology, University of UtahSalt Lake City, Utah, 84112; 2 Department of Entomology, Washington State UniversityPullman, Washington, 99164; Ecology and Evolutionary Biology, University of ArizonaTucson, Arizona, 85721

**Keywords:** Antibody, defense, ecoimmunology, *Geospiza fortis*, invasive species, *Philornis downsi*

## Abstract

Ecological immunology aims to explain variation among hosts in the strength and efficacy of immunological defenses. However, a shortcoming has been the failure to link host immune responses to actual parasites under natural conditions. Here, we present one of the first experimental demonstrations of a parasite-induced immune response in a wild bird population. The recently introduced ectoparasitic nest fly *Philornis downsi* severely impacts the fitness of Darwin's finches and other land birds in the Galápagos Islands. An earlier study showed that female medium ground finches (*Geospiza fortis*) had *P. downsi*-binding antibodies correlating with presumed variation in fly exposure over time. In the current study, we experimentally manipulated fly abundance to test whether the fly does, in fact, cause changes in antibody levels. We manipulated *P. downsi* abundance in nests and quantified *P. downsi-*binding antibody levels of medium ground finch mothers, fathers, and nestlings. We also quantified host behaviors, such as preening, which can integrate with antibody-mediated defenses against ectoparasites. *Philornis downsi*-binding antibody levels were significantly higher among mothers at parasitized nests, compared to mothers at (fumigated) nonparasitized nests. Mothers with higher antibody levels tended to have fewer parasites in their nests, suggesting that antibodies play a role in defense against parasites. Mothers showed no behavioral changes that would enhance the effectiveness of the immune response. Neither adult males, nor nestlings, had *P. downsi*-induced immunological or behavioral responses that would enhance defense against flies. None of the parasitized nests fledged any offspring, despite the immune response by mothers. Thus, this study shows that, while the immune response of mothers appeared to be defensive, it was not sufficient to rescue current reproductive fitness. This study further shows the importance of testing the fitness consequences of immune defenses, rather than assuming that such responses increase host fitness.

Host immune responses can protect against the negative fitness consequences of parasitism; however, the strength and effectiveness of these responses vary among hosts. Strong host immune responses are often assumed to correlate with greater host fitness. This study investigates the relationship between host immune response, parasite load, and host fitness using Darwin's finches and an invasive nest parasite. We found that while the immune response of mothers appeared defensive, it did not rescue current reproductive fitness.

## Introduction

Immune responses can protect hosts from the fitness costs of parasitism; however, the strength and effectiveness of immune-mediated defense varies among individuals. Variability has been linked to factors including, but not limited to, host reproductive condition (Horak et al. 26; Ilmonen et al. 31), stress (Lacoste et al. 34), evolutionary history of exposure (Lee and Klasing 35; Matson 37; Bonneaud et al. 4), and genetic factors (Beadell et al. 1). The ability to identify underlying causes of variation is limited by the context in which studies are performed (Graham et al. 23). A major challenge in ecological immunology has been drawing causal relationships between host immune responses and actual parasites, under natural conditions (Owen and Clayton 42; Owen et al. 44; Boughton et al. 5). Studies that experimentally manipulate parasite abundance and test for parasite-induced host immune responses have the potential to be very informative; unfortunately, such studies are few in number (Buechler et al. 6; De Coster et al. 11). In this article we report the results of one of the first such studies in a natural host–parasite system.

In the Galápagos Islands of Ecuador an introduced parasitic nest fly, *Philornis downsi*, has been implicated in recent population declines of several species of Darwin's finches (Dvorak et al. 17, 18; Wiedenfeld et al. 51; O'Connor et al. 40,c). Adult flies, which are not parasitic, lay their eggs in the nests of finches (Couri and Carvalho 10; Fessl et al. 19), or in the nares (nostrils) of nestlings (Galligan and Kleindorfer 22). Once the eggs hatch, the larvae live in the nest and feed on the blood of the nestling and adult female birds (Dudaniec et al. 13; Huber et al. 28). *Philornis downsi* is known to have a significant negative effect on the reproductive success of its hosts (reviewed in Koop et al. 33).

**Figure 1:**
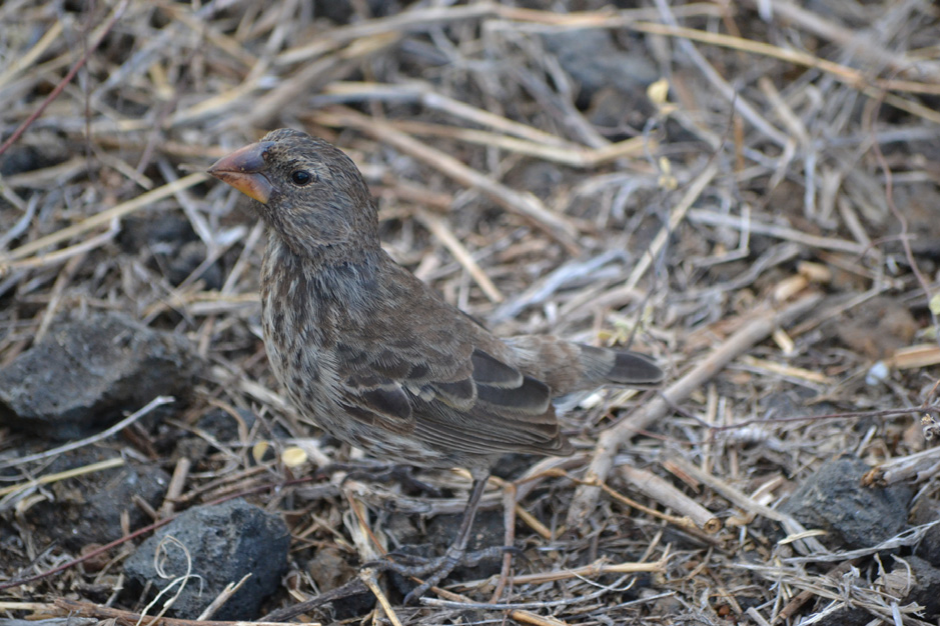
Photo of a female medium ground finch from El Garrapatero, Santa Cruz, Galapagos.

A recent study by Huber et al. (28) of medium ground finches (*Geospiza fortis*; Fig. 1) demonstrated increased levels of *P. downsi*-binding antibodies in birds during the nesting season, compared to birds sampled immediately prior to nesting. This increase in antibodies was observed in adult female birds, but not in adult males. Female finches incubate eggs and brood offspring, hypothetically increasing their exposure to *P. downsi* larvae in the nest. Male finches do not incubate eggs or brood nestlings. Although Huber et al. (28) showed a correlation between nesting and increased antibody level, this correlation could be driven by other variables such as immune stimulation induced by breeding stress (Pruett 46). An experimental manipulation of parasite abundance is needed to confirm the extent to which the immune response is actually caused by the parasite. To this end, we manipulated parasite abundance in nests to confirm that the observed changes in immune response are, in fact, induced by *P. downsi*, and are not the product of other temporal correlates.

We also monitored adult and nestling behavior with respect to *P. downsi* in the nest. Behavioral defense can be integrated with immune responses against ectoparasites (Lehane 36). For example, host antibodies produced against salivary proteins of ectoparasites are known to promote pruritus (itching), alerting the host to the presence of parasites (Wikel 52; Owen et al. 43). Hosts that respond to the presence of biting insects with defensive behaviors, such as preening, are far more likely to injure, kill, or reduce the feeding time of the parasite (Dusbabek and Skarkovaspakova 16; O'Connor et al. 39).

Yet another goal of this study was to investigate the role of immune responses in mitigating the fitness effects of *P. downsi*. Antibodies produced by hosts have the potential to act defensively against ectoparasites, like *P. downsi*, by facilitating the speed and intensity of inflammatory responses (Owen et al. 44). Inflammation of the skin inhibits blood feeding by preventing parasites from reaching host blood vessels with their mouthparts. Ectoparasites feeding on inflamed tissues may also ingest defensive peptides, or lytic molecules produced by the host that impair parasite feeding and digestion (Owen et al. 43). These components of the immune response can lead to dramatic reductions in the survival, development, and reproduction of parasites (Owen et al. 43). Thus, we compared the level of immune response by finches to the abundance of *P. downsi* larvae in their nests.

Finally, we quantified host reproductive success to investigate potential fitness consequences of host immune responses. Immune responses, even those associated with negative consequences for parasites, do not necessarily lead to increases in host fitness (Sheldon and Verhulst 49; Norris and Evans 38). Mounting an immune response is energetically expensive and may involve trade-offs with other fitness components, such as parental care or reproductive effort (Raberg et al. 47). Thus, hosts mounting strong immune responses against a parasite may have reduced fitness if they are less able to care for their offspring. Conversely, the benefit of reducing parasite abundance may outweigh the costs of an immune response and lead to a net increase in host fitness. Host immune response and behavior, parasite abundance, and host fitness must be measured simultaneously to rigorously interpret the influence of host immune defense on host fitness (Graham et al. 23).

## Material and Methods

### Site description and experimental design

The study was conducted during January–April 2010 on the island of Santa Cruz in the Galápagos Archipelago. Our field site, El Garrapatero, is a 1.5 × 1.5 km area in the arid, coastal zone. Medium ground finches are abundant at El Garrapatero, where they nest primarily in giant prickly pear cacti (*Opuntia galapageia*) (Huber 27). Clutch size ranges from 2 to 5 eggs, and females incubate for 10–14 days. Medium ground finch nestlings hatch asynchronously over a 2- to 4-day period. Nestlings spend 10–14 days in the nest, prior to fledging. In years of adequate food resources, medium ground finches can lay multiple clutches within a given breeding season, but they do not reuse the same nests (Grant 24).

We experimentally manipulated the abundance of *P. downsi* in medium ground finch nests and monitored host fitness following treatment. Additional factors, such as poor weather conditions, may have contributed to variation in host fitness; however, these effects were expected to act equally on across treatments. Therefore, by using an experimental approach, we were able to isolate and quantify the effect of only *P. downsi* on host fitness. To manipulate parasite abundance, we sprayed a 1% aqueous permethrin solution (Permectrin™ II, KMG-Bernuth, Inc., Houston, TX) into experimental nests; control nests were sham-fumigated with water. Nests were treated when the first nestling hatched, and again 4 days later. At each time period, nestlings and eggs were briefly removed from the nest along with a thin layer of nest material from the bottom of the nest. The nest was sprayed with either permethrin or water using a generic spray bottle with a fine mist setting. The nest was allowed to dry for several minutes at which point the nest material, nestlings, and eggs were returned to the nest (typically within 10 min of removal). Parents were quick to return to nests following treatment, and there were no cases of nest abandonment due to treatment. If a single pair of birds nested more than once during the study period, the treatment was reversed between reproductive bouts.

Active nests were visited every other day between 0600 and 1100 h to record the number of eggs and nestlings present. We continued to monitor nests until all nestlings died or fledged. Nestlings were marked shortly after hatching by coloring one toenail with a permanent marker (Sharpie^®^, Newell Rubbermaid, Oak Brook, IL). At ~5 days of age, nestlings were banded with a numbered monel metal band and three color bands. Successful fledging was confirmed by observing and identifying birds after they left the nest using color band combinations (Koop et al. 33). Once empty, nests were collected and placed in a sealed bag to quantify *P. downsi*.

### Parasite abundance

The 21 fumigated and 22 sham-fumigated nests were carefully dissected within 8 h of collection and any *P. downsi* larvae, pupae, and eclosed pupal cases were counted. First instar larvae can live subcutaneously in nestlings, making them impossible to quantify reliably. Therefore, total parasite abundance was the sum of all second and third instar larvae, pupae, and eclosed pupal cases found in the nest material or externally on nestlings. Larvae and pupae removed from nests were reared to confirm their identification as *P. downsi* (Dodge and Aitken 12).

### Blood sampling

When the oldest nestling was ~5 days old, we used a mist net to capture the parent birds near the nest between 0600 and 0900 h. From each parent we collected a small blood sample (90 μL) in a microcapillary tube via brachial venipuncture (*n* = 14 females and 10 males from fumigated nests, 15 females and 10 males from sham-fumigated nests). Adults were banded with a numbered monel metal band and three color bands before being released. We also collected a blood sample (30 μL) from each nestling when they were 5–6 days old via brachial venipuncture (*n* = 59 nestlings from fumigated nests, 10 nestlings from sham-fumigated nests). Samples were stored on wet ice in the field, then in a −20°C freezer at a field station, and ultimately in a −80°C freezer for longer term storage after the field season.

### Immunology

We used enzyme-linked immunosorbent assays (ELISA) to detect the presence of *P. downsi*-binding antibodies in the plasma of finches. Our protocol was modified slightly from that of Huber et al. (28). Briefly, 96-well plates were coated with 100 μL/well of *P. downsi* protein extract (capture antigen) diluted in carbonate coating buffer (0.05 mol/L, pH 9.6). Plates were incubated overnight at 4°C, then washed and coated with 200 μL/well of bovine serum albumin (BSA) blocking buffer and incubated for 30 min at room temperature on an orbital table. Between each of the following steps, plates were washed five times with a Tris-buffered saline wash solution, loaded as described, and incubated for 1 h on an orbital table at room temperature. Triplicate wells were loaded with 100 μL/well of individual finch plasma (diluted 1:500 in sample buffer). Plates were then loaded with 100 μL/well of Rabbit-αHOSP-IgY (primary detection antibody; diluted 1:10,000), followed by 100 μL/well of Goat-αRabbit-hrp (secondary detection antibody; diluted 1:20,000) (Bethyl Laboratories, Montgomery, TX). Finally, plates were loaded with 100 μL/well of peroxidase substrate (tetramethylbenzidine, TMB: Kirkegaard and Perry cat., Gaithersburg, MD, 50-77-03) and incubated for exactly 10 min. The reaction was stopped using 100 μL/well of 2 mol/L H_2_SO_4_. Optical density (OD) was measured using a spectrophotometer (BioTek, PowerWave HT, Winooski, VT, 450-nanometer filter).

On each plate, a positive control of pooled plasma was used in triplicate to correct for inter-plate variation. In addition, each plate contained a nonspecific binding (NSB) sample in which capture antigen, detection antibody, and secondary detection antibody were added, but plasma was excluded. Finally, each plate included a blank sample in which only the detection antibody was added, but plasma and capture antigen were excluded. NSB absorbance values were subtracted from the mean OD value of each sample. Antibody levels were compared between fumigated and sham-fumigated nests for adult females, adult males, and nestlings using a two-way analysis of variance (ANOVA) with Bonferroni multiple comparison post hoc tests (α < 0.05). We performed a Pearson correlation analysis to examine the relationship between female OD values from sham-fumigated nests and parasite abundance.

### Behavior

We monitored parental and nestling activities using battery-powered Sony^®^ (Tokyo, Japan) video camera systems. We placed small nest cameras (31 mm in diameter, 36 mm in length) in the tops of nests. The cameras were attached to small recording devices (PV700 Hi-res DVR, 8 × 12 × 3 cm, StuntCams, Grand Rapids, MI) hidden under brush. Behavior was recorded for ~3 h between 0600 and 1000 in haphazard subsamples of fumigated (*n* = 9) and sham-fumigated (*n* = 9) nests.

From the video recordings we quantified the amount of time males and females spent at the nest after nestlings hatched. We also quantified the following female behaviors, which were performed at the nest: feeding nestlings, nest sanitation, brooding nestlings, standing at the nest entrance, standing erect in the nest, self-preening, and allo-preening nestlings. All of these behaviors were mutually exclusive, with the exception of self-preening. Because females often preened while brooding nestlings, time spent self-preening was analyzed independently of other behaviors at the nest. All other behaviors are presented and analyzed as a proportion of time spent at the nest.

We also quantified the following male behaviors performed at the nest: feeding nestlings, nest sanitation, feeding the female, and standing at the nest entrance. Males do not brood nestlings. All of these male behaviors were mutually exclusive and are presented and analyzed as a proportion of the time observed at the nest. While at the nest, males were never observed preening themselves, or nestlings.

Time spent feeding nestlings was measured from the moment an adult began transferring food to a nestling until the adult's bill left contact with the last nestling. Nest sanitation was measured when an adult actively contacted the nest material with its bill (Christe et al. 8). Brooding time was measured when a female was sitting in the nest in direct contact with nestlings. Males and females also spent time standing at the nest entrance, but only females spent time standing erect inside the nest. Males performed all behaviors from the nest entrance; they were never observed entering the nest.

We also quantified two nestling behaviors: self-preening, defined as the amount of time a nestling moved its bill in contact with its body, and agitation, defined as shaking, repositioning, or jumping in the nest. Parents often blocked the camera, interfering with our ability to see nestlings; therefore, we only quantified nestling behavior when parents were not present at the nest. Nestling behaviors are reported as the proportion of the time they were observed without the parents present.

All videos were watched and scored by a single observer (M. A. A.) who was blind to nest treatment. Videos were analyzed using VLC media player (VideoLAN) and Quicktime 10.0 (Apple, Inc., Cupertino, CA). Nestlings in the videos ranged in age from 2 to 6 days and clutch size ranged from 1 to 5 nestlings. A single day of video for each nest was paired between treatments, based on nestling age and clutch size, such that neither mean nestling age, nor mean clutch size, differed significantly between treatments. We quantified behaviors from 54 total hours of video, with an average of 3 h for each of the 18 nests (nine fumigated, nine sham-fumigated). Two nests, one from each treatment, had males that were never observed at the nest while videotaping occurred. Therefore, we report behavior for females from 18 nests and for males from only 16 nests.

We used Wilcoxon matched-pair tests to compare the mean (±SE) time spent performing behaviors between treatments. We used Chi-square tests to compare the allocation of time across all behaviors performed at the nest. All statistical analyses were performed using in Prism^®^ v.5.0b (GraphPad Software, Inc., La Jolla, CA) or R v.2.12.2 (R Development Core Team, Vienna, Austria).

## Results

### Parasite abundance

**Figure 2:**
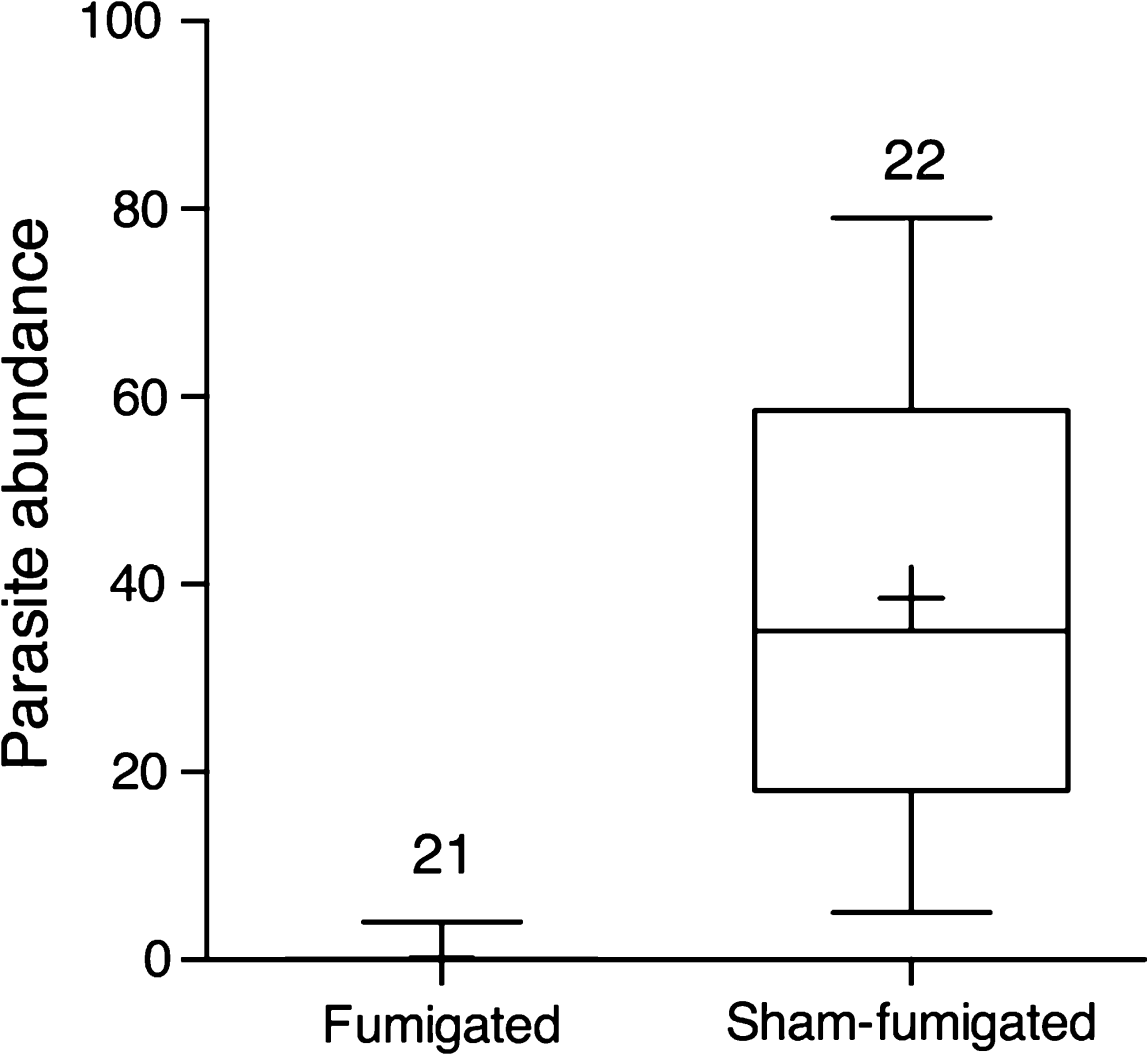
Box and whiskers plot of parasite abundance in fumigated and sham-fumigated nests. Boxes show the median, and the 25th% and 75th% for each treatment. Whiskers show the maximum and minimum values. The mean is indicated by a (+). The number of nests included in each treatment is shown above the bars.

The experimental treatment of nests with permethrin was effective in reducing parasite abundance. Sham-fumigated nests had a mean parasite abundance of 38.50 ± 5.13 *P. downsi*, compared to 0.23 ± 0.19 *P. downsi* in fumigated nests (*t* = 7.40, *P* < 0.0001; Fig. 2). Parasite abundance ranged from 5 to 79 parasites in sham-fumigated nests and from 0 to 4 parasites in fumigated nests. Nineteen fumigated nests were free of *P. downsi*; the remaining two nests, which experienced heavy rain soon after permethrin application, had very small numbers of *P. downsi* (one fly and four flies, respectively). *Philornis downsi* was found in all 22 sham-fumigated nests.

### Immunology

**Figure 3:**
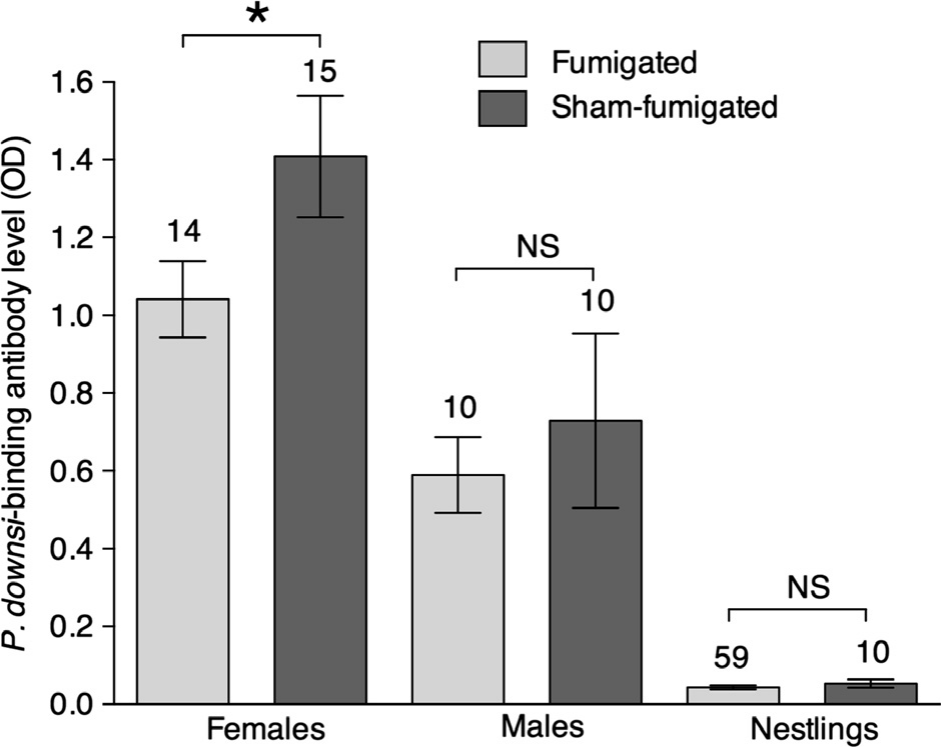
Mean (±SE) *Philornis downsi*-binding antibody response (optical density, OD) of adult females, adult males, and nestlings from fumigated and sham-fumigated nests. The number of individuals sampled is shown above each bar. Asterisk indicates a significant difference (*P* < 0.05) between treatments using Bonferroni post hoc comparisons (NS = nonsignificant).

*Philornis downsi*-binding antibody responses differed significantly with family status, that is among adult females, adult males, and nestlings (two-way ANOVA; family status: *F*_2, 105_ = 95.12, *P* < 0.001; Fig. 3). There was also a significant effect of nest treatment (treatment: *F*_1, 105_ = 5.18, *P* = 0.02), but no significant interaction between treatment and family status (treatment × family status: *F*_2, 105_ = 2.19, *P* = 0.12). Bonferroni post hoc multiple comparisons showed that females in sham-fumigated nests had significantly greater *P. downsi*-binding antibody levels than females in fumigated nests (*t* = 2.93, *P* < 0.05). However, neither male nor nestling antibody levels differed significantly between treatments (males: *t* = 1.02, *P* > 0.05; nestlings: *t* = 0.92, *P* > 0.05). Hence, only females showed a significant, detectable antibody response to the experimental manipulation of *P. downsi* abundance in nests.

**Figure 4:**
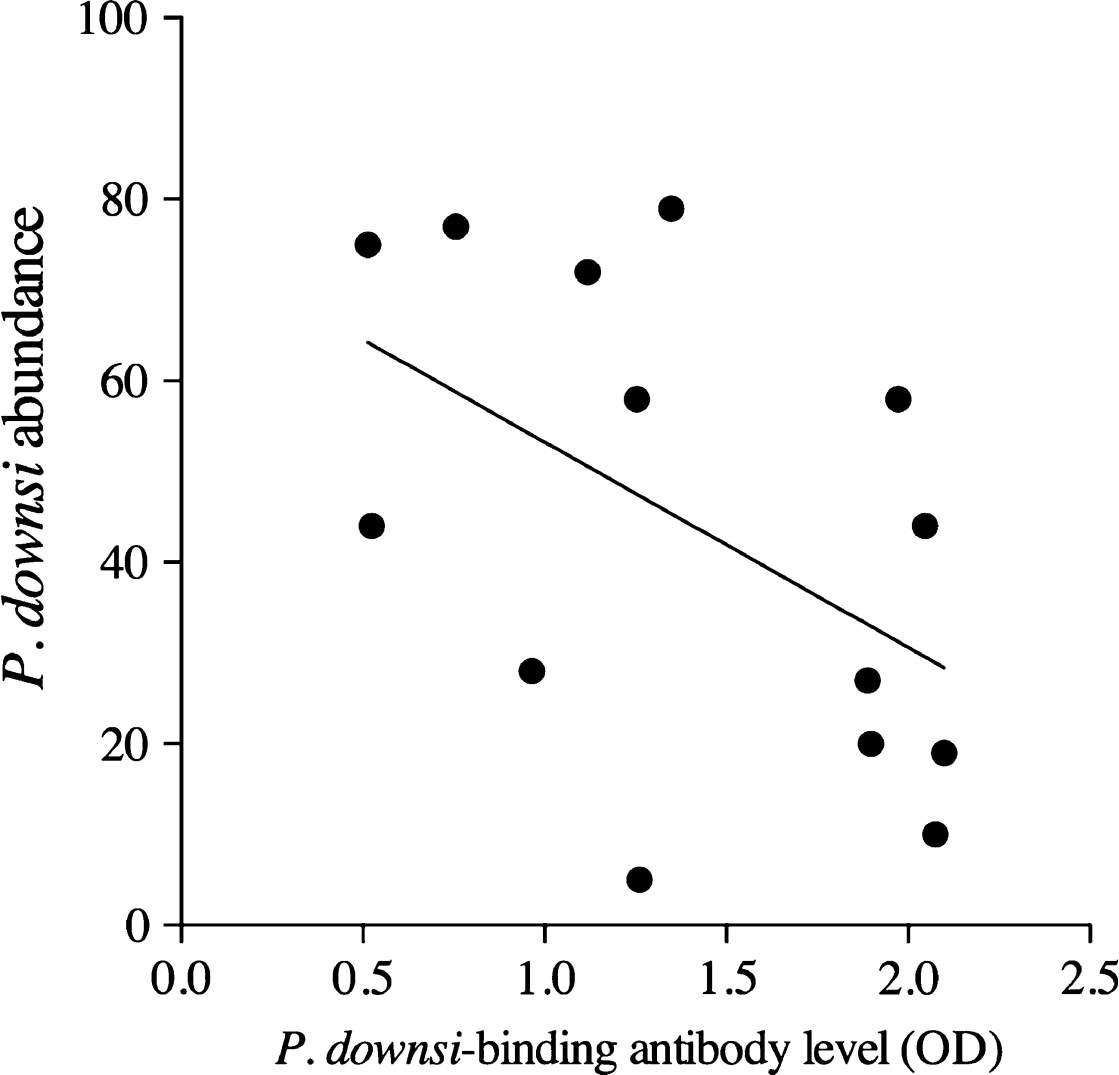
Relationship between adult female *Philornis downsi*-binding antibody level (optical density, OD) and *P. downsi* abundance in sham-fumigated nests. Each point represents a single female parent.

Among females from sham-fumigated nests (*n* = 14), *P. downsi*-binding antibody levels and parasite abundance were marginally correlated (Pearson correlation, *r* = −0.51, *P* = 0.06; Fig. 4). Females with greater *P. downsi*-binding antibody levels had fewer parasites in their nests.

### Behavior

Females did not differ significantly in the amount of time they spent at fumigated and sham-fumigated nests (*n* = 9 females from fumigated nests, nine females from sham-fumigated nests; Wilcoxon -matched pairs, *W* = −27.0, *P* = 0.13); females from fumigated nests spent 44.1 ± 6.6% of their time at the nest, compared to 56.1 ± 9.1% for females at sham-fumigated nests. Females spent very little time self-preening at the nest, and there was no significant effect of treatment on self-preening (*W* = 8.0, *P* = 0.59); females in fumigated nests spent only 1.9 ± 1.4% of their time preening, compared to 1.4 ± 1.0% for females in sham-fumigated nests. Females spent <1% of their time allo-preening nestlings, and there was no significant difference between treatments (*W* = −10.0, *P* = 0.13). Because preening was such an uncommon behavior, it was excluded from the additional comparisons of behavior below.

**Figure 5:**
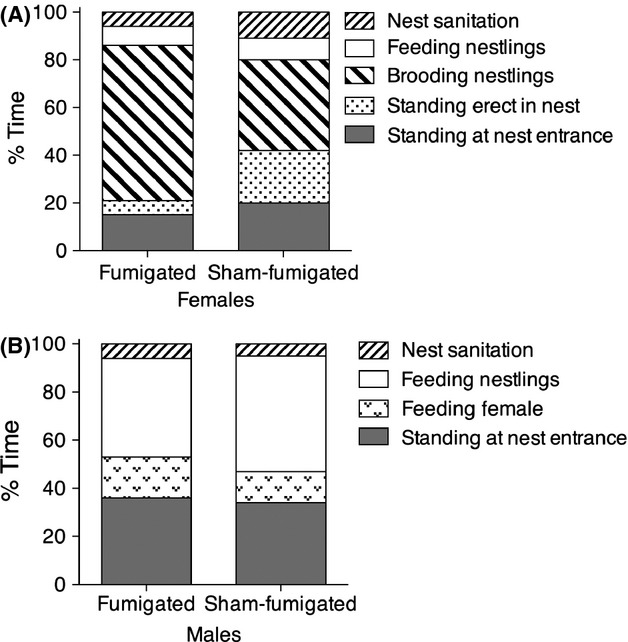
Mosaic plots of parental behaviors performed at the nest by (A) adult females and (B) adult males in fumigated and sham-fumigated nests (*N* = 9 nests per treatment for females; *N* = 8 nests per treatment for males).

Females differed significantly in the time they devoted to different behaviors at fumigated versus sham-fumigated nests (*χ*^2^ = 18.46, df = 4, *P* = 0.001, Fig. 5A). The largest difference between treatments was in the time females spent brooding their offspring, versus standing erect in the nest. Females in fumigated nests spent 65 ± 8.7% of their time brooding, compared to 38 ± 9.1% by females in sham-fumigated nests. Females in fumigated nests spent 5.7 ± 1.1% of their time standing erect in the nest, compared to 21.5 ± 6.3% by females in sham-fumigated nests.

Males did not differ significantly between treatments in the amount of time they performed different behaviors while at the nest (*n* = 8 males from fumigated nests, eight males from sham-fumigated nests; *χ*^2^ = 1.23, *P* = 0.75; Fig. 5B). Overall, males spent very little time at the nest and there was no significant difference between treatments in nest attendance by males (*W* = 29.0, *P* = 0.10). Males in fumigated nests spent 3.2 ± 0.8% of their time at the nest, while males in sham-fumigated nests spent 1.6 ± 0.4% of their time at the nest.

Nestlings in fumigated nests tended to show less agitation (0.2 ± 0.1%) than nestlings in sham-fumigated nests (1.3 ± 0.7%), though the difference was not statistically significant (*W* = −25.0, *P* = 0.09). However, the agitation behavior of nestlings in sham-fumigated nests was significantly more variable than that of nestlings in fumigated nests (range within sham-fumigated nests, 0–5.27%; range within fumigated nests, 0–0.64%; *F*-test to compare variances, *F* = 71.6, df = 8.8, *P* < 0.0001). In short, the variation in agitation by nestlings in sham-fumigated nests was eightfold more than that of nestlings in fumigated nests. Nestlings were observed preening in only three nests (one sham-fumigated, two fumigated). Nestlings spent <1% of their time self-preening, which did not differ significantly between treatments (*W* = −3, *P* = 0.50).

### Fledging success

All 21 fumigated nests (100%) fledged at least one offspring, while none of the 22 sham-fumigated nests (0%) fledged any offspring (Fisher's Exact, *P* < 0.0001). Fifty-six of 74 (76%) nestlings fledged from fumigated nests, compared to 0 of 62 (0%) nestlings from sham-fumigated nests (*P* < 0.0001).

## Discussion

This study experimentally demonstrates a parasite-induced immune response in a wild bird population. Our results show a causal link between a biologically relevant host immune response and an actual parasite, under natural conditions. Adult female, but not male, medium ground finches produced a significant immunological (antibody-mediated) response to *P. downsi*. Our results show experimentally that *P. downsi* does, in fact, stimulate an immune response in adult females, consistent with the correlation reported by Huber et al. (28). Furthermore, we show that females mounting stronger parasite-induced immune responses tend to have fewer parasites in their nests.

This study is one of the first demonstrations of an apparent effect of a parasite-induced immune response on parasite abundance in a wild bird. Work with other bird parasites has shown that antibody-mediated immune responses can increase the speed and intensity of the inflammatory response, preventing successful feeding of parasites and reducing parasite survival (Owen et al. 43). Alternatively, the mechanism could be indirect; for example, female antibody responses may promote itching that alerts the host to biting insects (Wikel 52; Owen et al. 43). Females that respond with defensive behaviors, such as preening, could kill, injure, or remove parasites (Dusbabek and Skarkovaspakova 16; O'Connor et al. 39). Further work is needed to explore additional variables that may be co-correlated with female immune response and parasite abundance.

A reduction in parasite burden is expected to benefit nestlings and thereby improve host reproductive success. However, within the parameters of this study, the observed decrease in parasite abundance did not help nestlings, as no nestlings fledged from any of the sham-fumigated nests. This result was surprising, given the results of a previous study performed in 2008 which showed that eradicating some, but not all, *P. downsi* from medium ground finch nests leads to increased fledging success (Koop et al. 33). Koop et al. (33) significantly reduced mean *P. downsi* abundance to ~21 parasites in nests treated with nest liners, compared to untreated nests, which had ~38 parasites per nest. This reduction was sufficient to increase fledgling success in lined nests, where 33% of nests fledged at least one offspring, compared to unlined nests, where only 4% of nests fledged any offspring. In this study, parasite abundance in sham-fumigated nests ranged from 5 to 79 *P. downsi* per nest (Fig. 2), yet no nestlings survived from nests in this treatment. The young age at which nestlings in this study died and the complete failure of nests even with low parasite abundance suggests that the impact of *P. downsi* on finches was unusually severe at our study site in 2010. One possible reason is that 2009 was a very dry year; annual rainfall in 2009 was 219 mm, compared to 503 mm in 2010 (Charles Darwin Foundation 7, Meteorological Database). Dry years reduce overall seed availability, meaning that the seed bank in 2010 may have been depleted (Schluter 48). Limited food resources are expected to negatively affect adult condition (Boag and Grant 2), which may have placed additional stress on nestlings.

Furthermore, annual differences in rainfall may have contributed to changes in *P. downsi* virulence. Multiple *P. downsi* females can infest a single finch nest and female flies can mate with multiple males (Dudaniec et al. 14). Thus, the relatedness between *P. downsi* larvae in a single finch nest has a relatively high degree of variability. Models of kin selection predict that when genetic relatedness of parasites is low, competition for within-host resources increases, leading to greater costs to the host (Frank 20, 21). While we did not collect data to directly test this idea, annual variation in climatic conditions may have altered the egg laying strategy of female flies, causing variation in parasite virulence between years. However, variation in parasite virulence could also be due to a number of other factors, such as host bird traits. Further investigation is needed to determine the role of biotic and abiotic factors on *P. downsi* virulence.

Independent of the effect on parasite abundance, female immune responses are thought to alter parental investment in current or future offspring (Raberg et al. 47; Bonneaud et al. 3). The ability of adult birds to perform parental behaviors can depend on the amount of energy invested (or not invested) in an immune response. Increases in nest sanitation and preening behaviors can serve to reduce parasite burden in the nest (Christe et al. 8; Hurtrez-Bousses et al. 30; Clayton et al. 9). Parents can also alter the rate at which they feed nestlings in order to provide energetic compensation for the direct negative effects of parasitism (Tripet and Richner 50; Hurtrez-Bousses et al. 29). Alternatively, birds can abandon nests with parasites in favor of future reproductive efforts (Duffy 15). O'Connor et al. (39) observed females of several finch species performing nest sanitation as well as allo-preening the feathers and nares of nestlings in nests with *P. downsi*. Interestingly, we observed almost no allo-preening; however, our observations were of younger nestlings (most of which were dead by 1 week of age). Our data show that while females did not abandon their parasitized nestlings or spend less time at the nest, they also did not significantly increase potentially beneficial behaviors, such as nest sanitation, or feeding nestlings. As in this study, O'Connor et al. (39) found no correlation between *P. downsi* intensity and parental feeding of nestlings.

Females in this study did, however, alter their brooding behavior; females in parasitized nests brooded significantly less and stood up more than females in fumigated nests. Whether this behavior was in response to agitated nestlings, or the parasites themselves, standing was probably an avoidance strategy for females (Hart 25). Although this study shows that these responses were not sufficient to rescue current reproduction, further study is needed to determine whether female responses increase their ability to invest in future reproduction.

Young altricial nestlings are expected to serve as primary hosts for nest parasites because they lack the necessary motor skills to preen or stand. Furthermore, both the innate and acquired arms of the immune system are developing in nestlings, perhaps making them incapable of mounting a robust immune response to parasites (Palacios et al. 45). We found no detectable difference in antibody levels of nestlings in fumigated and sham-fumigated nests. This result suggests that nestlings are not able to defend themselves immunologically against *P. downsi* in the nest. However, it should be noted that the rapid mortality of nestlings in sham-fumigated nests limited our sampling to young nestlings (~5 days of age). A recent study by King et al. (32) found that nestlings of some species of birds can start producing parasite-induced antibodies endogenously within 3–6 days of age. Thus, quantification of antibodies from older nestlings (6–14 days old) may yield different results. Of course, the ability of nestlings to produce *P. downsi*-binding antibodies and immunologically defend themselves against nest parasites is dependent upon their survival to that time point.

Our results suggest that young nestlings are incapable of responding behaviorally to *P. downsi*. O'Connor et al. (39) observed medium ground finch nestlings preening themselves in a nest parasitized by *P. downsi*. The same nestlings were also observed trying to climb on top of one another, possibly to escape *P. downsi* larvae attempting to feed. Nestlings in this study were observed preening only rarely (<1% of time), and the behavior did not differ significantly between treatments. Nestlings from sham-fumigated nests tended to show more agitated behavior than those in fumigated nests. Periods of agitation included shaking and repositioning within the nest, but we did not observe nestlings standing on top of one another. Again, many of the nestlings observed by O'Connor et al. (39) were significantly older (>8 days of age), which may explain the differences in behavior between studies. Very young nestlings lack the necessary motor skills to preen themselves, or stand.

Studies that explore trade-offs between host immune response and life-history components often operate under the assumption that stronger immune responses are positively correlated with higher fitness (Norris and Evans 38). This study demonstrates immunological activity of birds in response to a biologically relevant parasite. The data further suggest that stronger immune responses are defensive, because higher antibody levels are marginally correlated with lower parasite abundance. However, higher antibody levels did not result in higher reproductive success. This study provides a cautionary tale: even when stronger immune responses lead to lower parasite load, this does not necessarily result in higher host fitness. Our study underscores the importance of studying interactions between the host immune system, parasite load, and host fitness in order to derive robust conclusions regarding the functional significance of the immune system in an ecological context (Owen et al. 44; Graham et al. 23).
